# U-shaped association between the triglyceride–glucose index and atrial fibrillation incidence in a general population without known cardiovascular disease

**DOI:** 10.1186/s12933-023-01777-9

**Published:** 2023-05-18

**Authors:** Xiao Liu, Ayiguli Abudukeremu, Yuan Jiang, Zhengyu Cao, Maoxiong Wu, Jianyong Ma, Runlu Sun, Wanbing He, Zhiteng Chen, Yangxin Chen, Peng Yu, Wengen Zhu, Yuling Zhang, Jingfeng Wang

**Affiliations:** 1 Department of Cardiology, Sun Yat-Sen Memorial Hospital of Sun Yat-Sen University, Guangzhou, China; 2 Guangdong Provincial Key Laboratory of Malignant Tumor Epigenetics and Gene Regulation, Guangdong-Hong Kong Joint Laboratory for RNA Medicine, Sun Yat-Sen Memorial Hospital of Sun Yat-Sen University, Guangzhou, China; 3 Guangdong Province Key Laboratory of Arrhythmia and Electrophysiology, Guangzhou, China; 4 Institute for the Study of Endocrinology and Metabolism in Jiangxi Province, The Second Affiliated Hospital of Nanchang University, Jiangxi, China; 5 Department of Pharmacology and Systems Physiology, University of Cincinnati College of Medicine, Cincinnati, USA; 6 Department of Endocrine, The Second Affiliated Hospital of Nanchang University, Jiangxi, China; 7 Department of Cardiology, The First Affiliated Hospital of Sun Yat-Sen University, Guangzhou, China

**Keywords:** TyG index, Insulin resistance, Atrial fibrillation, ARIC study, General population

## Abstract

**Objective:**

The triglyceride–glucose (TyG) index has been shown to be a new alternative measure for insulin resistance. However, no study has attempted to investigate the association of the TyG index with incident atrial fibrillation (AF) in the general population without known cardiovascular diseases.

**Methods:**

Individuals without known cardiovascular diseases (heart failure, coronary heart disease, or stroke) from the Atherosclerosis Risk in Communities (ARIC) cohort were recruited. The baseline TyG index was calculated as the Ln [fasting triglycerides (mg/dL) × fasting glucose (mg/dL)/2]. The association between the baseline TyG index and incident AF was examined using Cox regression.

**Results:**

Of 11,851 participants, the mean age was 54.0 years; 6586 (55.6%) were female. During a median follow-up of 24.26 years, 1925 incidents of AF cases (0.78/per 100 person-years) occurred. An increased AF incidence with a graded TyG index was found by Kaplan‒Meier curves (*P* < 0.001). In multivariable-adjusted analysis, both < 8.80 (adjusted hazard ratio [aHR] = 1.15, 95% confidence interval [CI] 1.02, 1.29) and > 9.20 levels (aHR 1.18, 95% CI 1.03, 1.37) of the TyG index were associated with an increased risk of AF compared with the middle TyG index category (8.80–9.20). The exposure-effect analysis confirmed the U-shaped association between the TyG index and AF incidence (*P* = 0.041). Further sex-specific analysis showed that a U-shaped association between the TyG index and incident AF still existed in females but not in males.

**Conclusions:**

A U-shaped association between the TyG index and AF incidence is observed in Americans without known cardiovascular diseases. Female sex may be a modifier in the association between the TyG index and AF incidence.

**Graphical Abstract:**

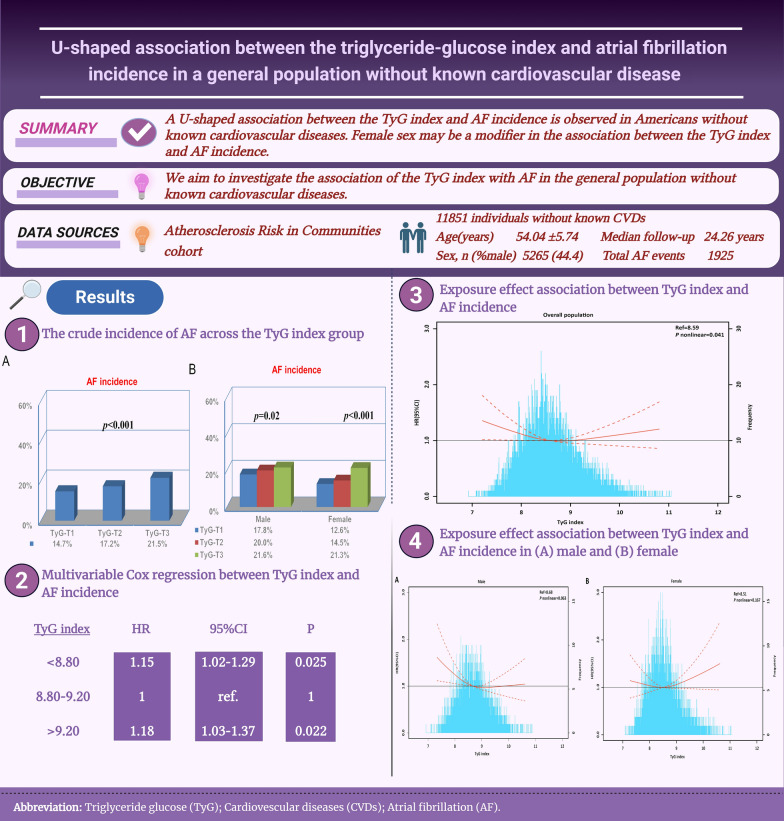

**Supplementary Information:**

The online version contains supplementary material available at 10.1186/s12933-023-01777-9.

## Background

Atrial fibrillation (AF) is the most prevalent sustained arrhythmia in clinical practice and is associated with high morbidity and mortality [1]. According to previous statistics, the estimated global prevalence of AF was 46.3 million [2, 3], and by 2050, the number of individuals with AF is estimated to reach 6–16 million in America, which indicates a higher burden of AF-relevant events. There are several risk factors for AF, such as hypertension, obesity, and diabetes. A recent study showed that lower high-density lipoprotein cholesterol, higher blood pressure, abdominal obesity, and higher fasting glucose are positively associated with the risk of AF [4]. As the main feature of type 2 diabetes mellitus (T2DM), insulin resistance (IR) are suspected to be a risk factor for AF. There is evidence that in a nondiabetic individual-based prospective cohort study, a positive association between IR and the risk of AF is found (N = 8175, hazard risk = 1.61) [5]. Individuals with IR are also found to be at higher risk of AF recurrence after catheter ablation [6]. Homeostatic model assessment (HOMA), as the “gold standard” method for the evaluation of insulin sensitivity, is not widely used in clinical practice because of the costly and time-consuming nature of the process^11^. Recent studies have shown that a novel measure, the triglyceride glucose (TyG) index, is useful for estimating IR, with the advantages of simplicity and wide accessibility [7, 8]. In addition, evidence from epidemiological studies showed that the TyG index may be advantageous for predicting IR risk compared with HOMA-IR [9–11].

Previous studies [12–29] and our recent work [31, 30] have reported a significant association between the TyG index and the incidence of atherosclerosis, myocardial infarction (MI), stroke, coronary artery disease and mortality in the general population regardless of T2DM status. Furthermore, the TyG index is associated with adverse cardiovascular events among diabetic or nondiabetic patients with established cardiovascular diseases (CVDs) [32–38]. However, no study has focused on the impact of the TyG index on AF incidence in the general population. Accordingly, in this study, by using the dataset from the Atherosclerosis Risk in Communities (ARIC) cohort, we aim to investigate the association between the TyG index and the incidence of AF in individuals without known CVDs.

## Methods

### Study database

This study is a sub-analysis of the ARIC study [39]. The ARIC study, a prospective cohort aiming to (1) investigate the etiology and natural history of atherosclerosis, (2) investigate the etiology of clinical atherosclerosis diseases, and (3) measure variation in cardiovascular risk factors, medical care, and disease by race, sex, place, and time [39]. The ARIC study sampled 15,792 individuals aged 45 to 64 from 4 US communities. To date, it has finished 7 visits, which include a baseline examination (visit 1), visit 2 (occurring in 1990–1992), visit 3 (occurring in 1993–1995), visit 4 (occurring in 1996–1998), visit 5 (occurring in 2011–2013), visit 6 (occurring in 2016–2017) and visit 7 (occurring in 2018–2019). In this study, we included participants with AF-relevant information at visit 5. The ARIC study complied with the Declaration of Helsinki, and all participants signed informed consent before randomization. The study design, baseline characteristics, and major results have been published previously [39, 40].

### Definitions of the TyG index and study outcomes

The baseline TyG index was calculated as the Ln[fasting triglycerides (mg/dL) × fasting glucose (mg/dL)/2] [9]. Blood samples were obtained after fasting for 8 h or more and tested in the central clinical chemistry laboratory and central lipid laboratory. Fasting plasma glucose and fasting triglyceride levels were measured via the optimized dart glucose reagent method and enzymatic method, respectively. In ARIC study, the process of sample collection to test was monitored using internal and external quality control.

The interested outcome was AF incidence, which was defined in compliance with the ARIC study: the studied individuals (1) had a fatal AF event; (2) had an incident AF event at visit 2, 3, 4, or 5, which was determined by electrocardiogram (ECG) readings; and (3) had an incident AF event as determined by hospital discharge codes on the cohort eligibility form [41].

### Clinical and laboratory measurements

Demographic, clinical, and comorbidity data were recorded. Smoking status was classified as never smoking, past smoking, or current smoking; drinking status was also classified as never drinking, past drinking, or current drinking. Physical activity was assessed by the Baecke Sport Activity Score, which is a sport index of leisure time during the year and ranges from 1 to 5 (a higher score indicates a higher intensity). The Baecke Sport Activity Score was determined by summing the scores from four different items: (1) the sport activity recorded by them (intensity in Mj/h, duration in hours per week, and proportion of the year the activity performed); (2) asking the individuals to rate their physical activity by comparing with others (much less, less, the same, more, much more); (3) how often the participants sweat (never, seldom, sometimes, often, very often); and (4) how often the participants play sports or exercises (never, seldom, sometimes, often, very often) [42, 43]. Body mass index (BMI) was calculated as weight in kilograms divided by height in meters squared. Low-density lipoprotein-cholesterol (LDL-C) levels were determined by the Friedewald formula and high-density lipoprotein-cholesterol (HDL-C) levels were determined using standardized enzymatic assays. Cholesterol-lowering medication was determined based on the previous two weeks’ self-reported usage report or medications brought by participants to the visit. Seated blood pressure was recorded by the mean of the last two of three measurements (random-zero sphygmomanometer) after a 5-min seat. Hypertension was defined as systolic blood pressure readings ≥ 140 mmHg, or diastolic blood pressure readings ≥ 90 mmHg, or the use of antihypertensive drugs in the previous two weeks. Peripheral artery disease (PAD) was defined according to the ankle-brachial index (ABI). If ABI04 < 0.90, the participant was diagnosed with PAD. Diabetes was defined as a fasting glucose level ≥ 126 mg/dl or nonfasting glucose level ≥ 200 mg/dl, self-reported physician diagnosis of diabetes, or any use of antidiabetic drugs. Heart failure was defined according to the use of any medication for heart failure or qualifies for the relevant criteria. Coronary heart disease and stroke were defined according to participants’ self-reports or relevant measurements [39, 44, 45]. CVDs included the presence of heart failure, coronary heart disease, or stroke.

### Data analyses

The baseline characteristics of patients stratified by the TyG index category were compared. Continuous normally distributed data variables are expressed as the means with standard deviations (SDs), while nonnormally distributed data are expressed as medians with interquartile ranges (IQRs). The normality of the data was analyzed using the Shapiro‒Wilk test. For continuous variables, the differences between the groups were compared using unpaired Student’s *t* tests between 2 groups and one-way ANOVA among 3 groups (normal distribution) or Mann‒Whitney *U* tests between 2 groups and Kruskal–Wallis tests among 3 groups (nonnormal distributions). The categorical variables were reported as counts and percentages and compared using *χ*^2^ tests. The TyG index was categorized into three groups according to the shape of the exposure‐effect relationship, with the lowest level being regarded as the reference group (8.80–9.20). The Kaplan‒Meier method was used for incidence curve estimation. The association of the TyG index with incident AF was calculated using a univariate or multivariate Cox proportional hazard model, which is expressed by hazard ratios (HRs) and 95% confidence intervals (CIs). The proportional hazards assumption was satisfied by the TyG index (P = 0.26). The adjustments were selected based on a well-established prognostic significance: age, sex, BMI, race, smoking status, drinking status, LDL-C, HDL-C, use of cholesterol-lowering medication, and diseases at baseline, including hypertension, diabetes mellitus, and PAD. The exposure‐effect associations between the TyG index and incident AF were evaluated by using a restricted cubic spline function with 3 knots (10th, 50th, and 90th percentiles) [46]. For sensitivity analysis, we added physical activity and glucose lowering medication to the fully adjusted variables or restricted the study population to a nondiabetic population. SPSS Statistics Version 25.0 (IBM SPSS Statistics, IBM Corporation, Chicago, IL, USA) and the R Programming Language (Version 4.2.0) were used to run the statistical analysis. *P* ≤ 0.05 was considered to be statistically significant in all analyses.

## Results

**Fig. 1:**
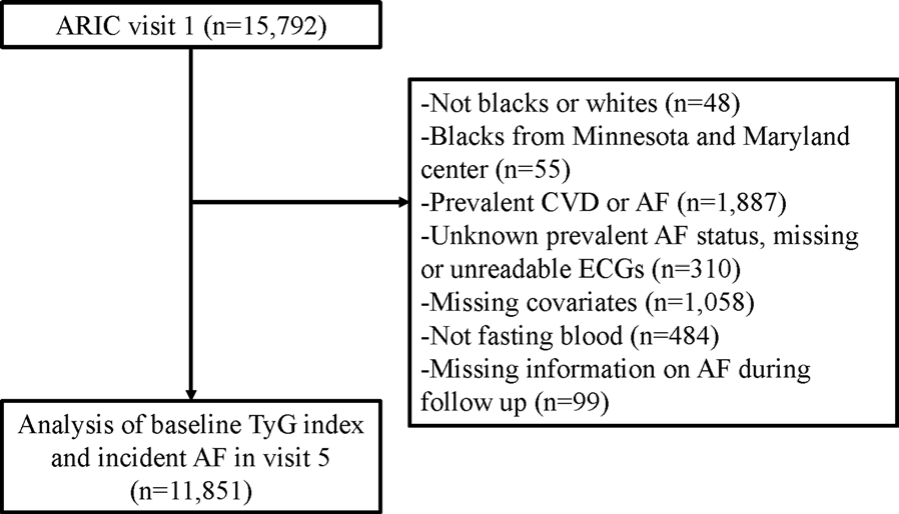
The following chart of study subject enrollment in the study of association between TyG index and atrial fibrillation incidence. *ARIC study* Atherosclerosis Risk in Communities, *TyG index* triglyceride–glucose index, *AF* atrial fibrillation, *CVD* Cardiovascular disease, *ECG* electrocardiogram

In total, 15,792 subjects were recruited for the ARIC study. After excluding individuals with a race other than African American or Caucasian and the few African American participants from Minneapolis and Washington County (n = 103), with baseline CVD or AF (n = 1887), with unknown prevalent AF status, missing or unreadable ECGs (n = 310), with missing covariates (n = 1058), with no fasting blood sample (n = 484) and missing information on AF during follow-up (n = 99), our study included 11,851 individuals without known CVDs (Fig. 1).

### Baseline characteristics of study participants

Baseline characteristics are listed in Table 1. Of 11,851 participants without known CVDs, the mean age was 54.04 (SD: 5.74) years; 6586 (55.6%) were female; 9078 (76.6%) were Caucasian; mean BMI was 27.37 (SD: 5.15) kg/m^2^; 1059 (8.9%) and 3633 (30.7%) had a history of diabetes and hypertension, respectively; and 286 (2.4%) used cholesterol-lowering medication. Participants were divided into three groups based on the baseline TyG index levels (Group 1: TyG index < 8.80; Group 2: 8.80 ≤ TyG index ≤ 9.20; and Group 3: TyG index > 9.20). The three groups were significantly different in terms of age, sex, BMI, race, smoking status, history of clinical diabetes, hypertension, PAD, cholesterol-lowering medication use, physical activity, and levels of HDL-C and LDL-C.

**Table 1:** Baseline characteristics of included subjects stratified by baseline TyG index

Variables	Full Eligible Cohort	TyG index			P-value
		< 8.80	8.80–9.20	> 9.20	
n	11,851	7605	2477	1769	
Age(years)	54.04 ± 5.74	53.61 ± 5.73	54.65 ± 5.75	55.08 ± 5.54	< 0.001
Sex, n (% male)	5265 (44.4)	3066 (40.3)	1236 (49.9)	963 (54.4)	< 0.001
BMI (kg/m^2^)	27.37 ± 5.15	26.47 ± 5.01	28.54 ± 5.05	29.61 ± 4.91	< 0.001
Race, n (%)					< 0.001
Caucasian	9078 (76.6)	5612 (73.8)	2036 (82.2)	1430 (80.8)	
African American	2773 (23.4)	1993 (26.2)	441 (17.8)	339 (19.2)	
Smoking status, n (%)					< 0.001
Current smoker	2992 (25.2)	1913 (25.2)	630 (25.4)	449 (25.4)	
Previous smoker	3778 (31.9)	2295 (30.2)	832 (33.6)	651 (36.8)	
Never smoked0	5081 (42.9)	3397 (44.7)	1015 (41.0)	669 (37.8)	
Drinking, n (%)					0.615
Current	6901 (58.2)	4442 (58.4)	1431 (57.8)	1028 (58.1)	
Previous	2071 (17.5)	1300 (17.1)	445 (18.0)	326 (18.4)	
Never	2879 (24.3)	1863 (24.5)	601 (24.3)	415 (23.5)	
Baecke sport activity score^a^	2.45 ± 0.80	2.47 ± 0.81	2.44 ± 0.77	2.39 ± 0.78	0.001
History of clinical DM, n (%)	1059 (8.9)	235 (3.1)	207 (8.4)	617 (34.9)	< 0.001
History of hypertension	3633 (30.7)	1907 (25.1)	882 (35.6)	844 (47.7)	< 0.001
History of PAD	442 (3.7)	261 (3.4)	104 (4.2)	77 (4.4)	0.07
Glucose (mg/dL)	105.04 ± 30.94	97.38 ± 10.91	105.54 ± 20.09	137.26 ± 63.62	< 0.001
Triglycerides (mg/dL)	123.02 ± 63.38	86.88 ± 25.08	154.80 ± 25.23	233.91 ± 63.14	< 0.001
HDL-C (mmol/L)	1.35 ± 0.44	1.48 ± 0.44	1.18 ± 0.34	1.05 ± 0.30	< 0.001
LDL-C (mmol/L)	3.55 ± 1.00	3.40 ± 0.97	3.83 ± 0.98	3.77 ± 1.06	< 0.001
TyG index	8.63 ± 0.56	8.30 ± 0.32	8.98 ± 0.12	9.57 ± 0.32	< 0.001
Cholesterol lowering medication, n (%)	286 (2.4)	136 (1.8)	70 (2.8)	80 (4.5)	< 0.001
Glucose lowering medication, n (%)^**b**^	380 (3.2)	68 (0.9)	60 (2.4)	252 (14.2)	< 0.001

*BMI* Body-mass index, *DM* diabetes mellitus, *PAD* peripheral artery disease, *HDL-C* high-density lipoprotein cholesterol, *LDL-C* low-density lipoprotein cholesterol, *TyG index* triglyceride–glucose index

^a^Values available in 11813 participants

^**b**^Values available in 11844 participants

### TyG index and incident AF

**Fig. 2:**
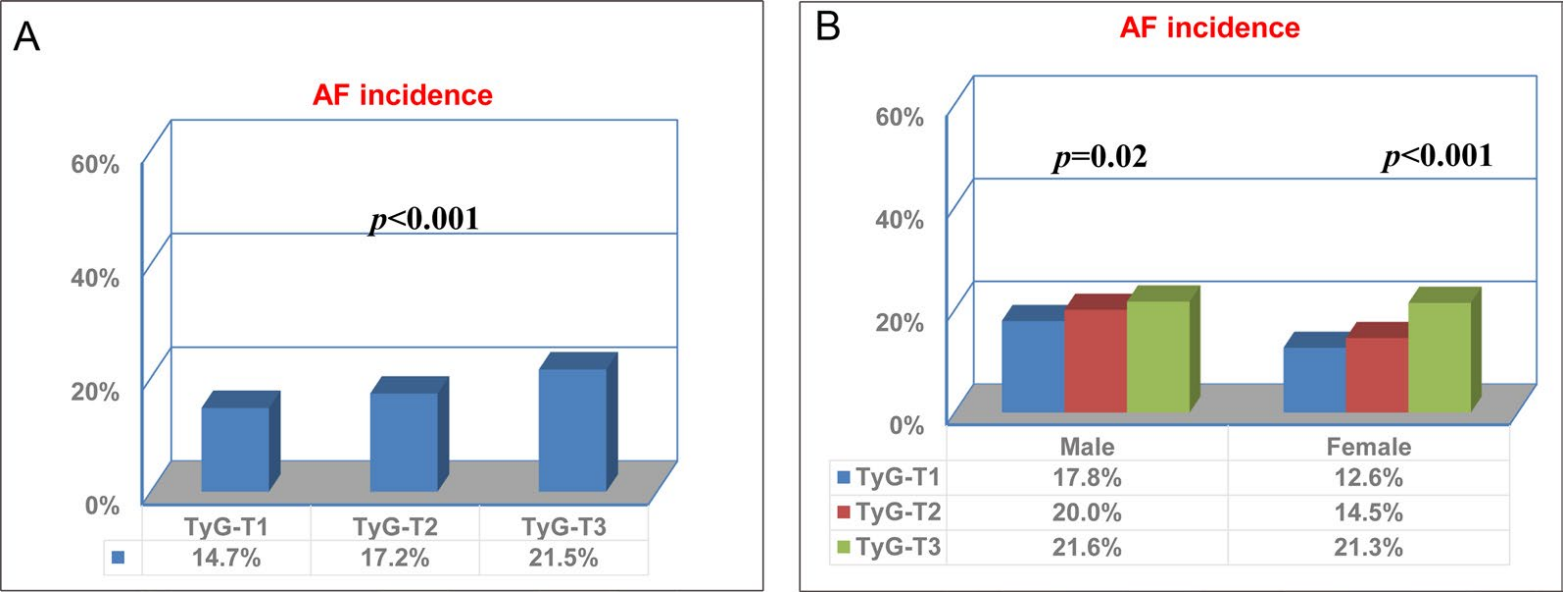
**A** The crude incidence of AF across TyG index group among overall individuals. **B** The crude incidence of AF across TyG index group basing sex subgroup. TyG-T1 versus TyG-T2 P < 0.001; TyG-T2 versus TyG-T3 P < 0.002; TyG-T1 versus TyG-T3 P < 0.001. TyG-T1: < 8.80, TyG-T2: 8.80–9.20, TyG-T3: > 9.20. *TyG index* triglyceride–glucose index. *AF* atrial fibrillation

**Fig. 3:**
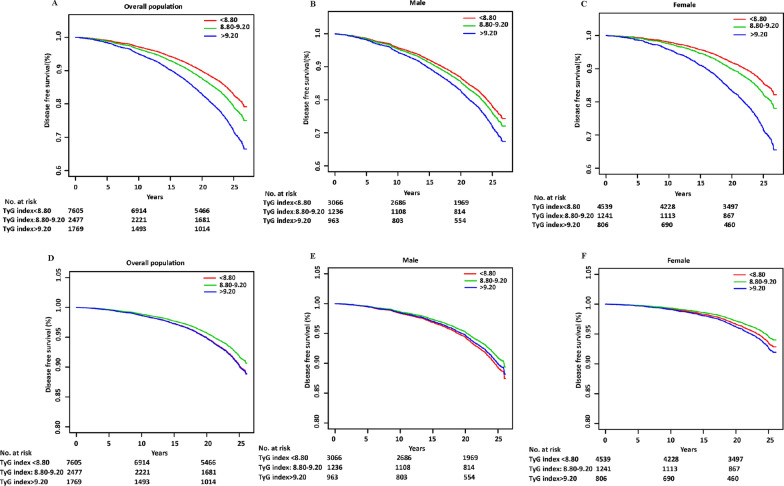
**A** Kaplan–Meier analysis for AF among TyG index groups in (**A**) all population, (**B**) male, and (**C**) female. AF-free survival was analyzed by a log-rank test (P < 0.001 in all populations, P = 0.002 in males, P < 0.001 in females). Adjusted incidence curve for AF among TyG index groups in (**D**) all population, (**E**) male and (**F**) female. The adjustments were age, sex, race, body mass index, smoking, drinking, low-density lipoprotein-cholesterol, high-density lipoprotein-cholesterol, use of cholesterol-lowering medication, history of diabetes, hypertension, and peripheral artery disease. *AF* atrial fibrillation. *TyG index* triglyceride–glucose index

**Fig. 4:**
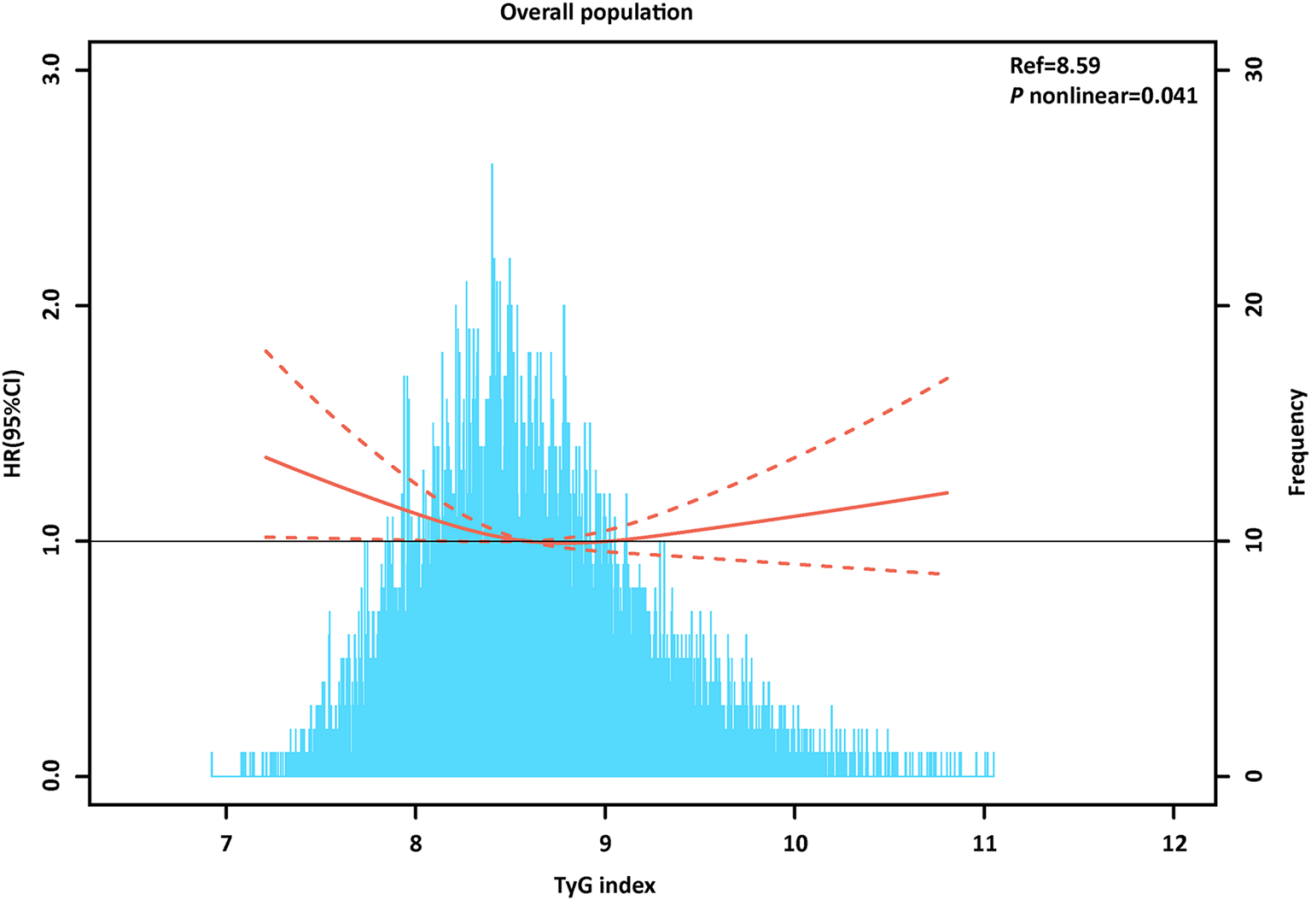
Multivariable-adjusted hazard ratios for AF based on restricted cubic spines among overall individuals. Each hazard ratio was computed with a TyG index level of 8.59 as the reference. The model was adjusted for age, sex, race, body mass index, smoking, drinking, low-density lipoprotein-cholesterol, high-density lipoprotein-cholesterol, use of cholesterol-lowering medication, history of diabetes, hypertension, and peripheral artery disease. The Red line represents references for HRs, and the area between the dotted line represents 95% CI. Histograms represent the frequency distribution of the baseline TyG index. *AF* atrial fibrillation. *HR* hazard ratio, *CI* confidence interval *TyG* triglyceride–glucose

During 246,908.0 person-years of follow-up (median follow-up of 24.3 years), 1,925 incident cases of AF occurred. The crude incidence of AF across the TyG index group is shown in Fig. 2A, which shows an increased AF incidence with the graded TyG index (*P* < 0.05) in individuals without known CVDs. Similar results were found by the Kaplan‒Meier curves (*P* for log-rank test < 0.001) in Fig. 3A. Interestingly, the adjusted incidence curve showed the lowest AF incidence in the middle TyG category (8.80–9.20), whereas a similar risk was observed with the first (< 8.80) and third (> 9.20) TyG groups (Fig. 3D). Consistently, in Cox proportional hazard analysis, the unadjusted model showed a graded incidence of AF compared with the middle TyG index. However, both the first TyG index group (HR: 1.15, 95% CI 1.02 to 1.29) and third TyG index group (HR 1.18; 95% CI 1.03 to 1.37) showed an increased incidence compared with the middle TyG index group after adjustment for age, sex, race, body mass index, smoking, drinking, low-density lipoprotein-cholesterol, high-density lipoprotein-cholesterol, use of cholesterol-lowering medication, history of diabetes, hypertension, and PAD (Table 2). The restricted spline model confirmed a U shape between the TyG index and AF incidence in individuals without known CVDs (*P* = 0.041) (Fig. 4).

**Table 2:** Cox proportional hazards analysis evaluating prognostic implication of categorical TyG index for AF

TyG index	AF event/total	Person-years	Incidence rate (Per 100 person-years)	Unadjusted HR (95% CI)	P-value	Adjusted HR (95% CI)	P-value
< 8.80	1118/7605	161,815.85	0.69	0.81 (0.72, 0.91)	< 0.001	1.15 (1.02,1.29)	0.025
8.80–9.20	427/2477	51,327.01	0.83	1(ref.)	1	1(ref.)	1
> 9.20	380/1769	33,765.13	1.13	1.42 (1.23, 1.63)	< 0.001	1.18 (1.03,1.37)	0.022

Group1-group3 were stratified basing TyG index: group1: < 8.80, group2: 8.80–9.20, group3: > 9.20. HR adjusted for age, sex, race, body mass index, smoking, drinking, low density lipoprotein-cholesterol, high density lipoprotein-cholesterol, use of cholesterol lowering medication, history of diabetes, hypertension, peripheral artery disease

*AF* atrial fibrillation, *TyG index* triglyceride–glucose index, *HR* hazard ratios, *CI* confidence intervals

### Subgroup analysis

The baseline characteristics of the participants stratified by sex are also shown in Additional file 1: Table S1. The females were more likely to be nonsmokers, nondrinkers, and African American and showed significantly lower age, LDL-C, TyG index, glucose, triglycerides, and physical activity while they had higher HDL-C, cholesterol-lowering medication use, and a history of PAD.

**Fig. 5:**
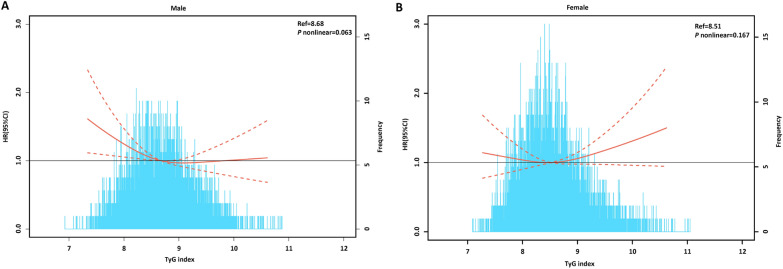
Multivariable-adjusted hazard ratios for AF based on restricted cubic spines in **A** male and **B** female. Each hazard ratio was computed with a TyG index level of **A** 8.68 and **B** 8.51 as the reference. Model was adjusted for age, race, body mass index, smoking, drinking, low density lipoprotein-cholesterol, high density lipoprotein-cholesterol, use of cholesterol lowering medication, history of diabetes, hypertension, peripheral artery disease. Red line represents references for HRs, and area between dotted line represent 95% CI. Blue area represents the fraction of the population with different baseline TyG index. *HR* hazard ratio, *CI* confidence interval, *TyG* triglyceride–glucose, *AF* atrial fibrillation

We further explored the sex-specific association of the TyG index with AF incidence, which is shown in Fig. 2B. An increased crude AF incidence with TyG index grades was observed, either in males or females. Moreover, the Kaplan‒Meier curves also showed a grade-increased AF incidence with an increased TyG index (*P* = 0.002 in males, *P* < 0.001 in females) (Fig. 3B, C). Interestingly, the adjusted incidence curve showed the lowest AF incidence in the middle TyG category (8.80–9.20) in both sexes. However, the AF incidence in the first TyG category (< 8.80) was higher than that in the third TyG category (> 9.20) in males, while the opposite was true in females (Fig. 3E, F). In further exposure‐effect analysis, a U shape of the dose–response curve was seen in females (*P* = 0.167). For males, the curve increased steeply in the lower TyG index group but was much flatter in the TyG index > 9.20 group (*P* = 0.063) (Fig. 5A, B).

### Sensitivity testing

First, as we previously showed physical activity is associated with a reduced incidence of AF [47]. To account for physical activity, we evaluated the TyG index and incidence of AF after additionally adjusting for physical activity. A U-shaped association of the TyG index with the incidence of AF reminded (Additional file 1: Table S2). Second, with adjustments further applied by glucose-lowering medication, a U-shaped association remained confirmatory (Additional file 1: Table S2). Third, we restricted the study population to nondiabetic individuals. This Cox regression sensitivity analysis also showed a trend of U-shaped association with the TyG index of AF (Additional file 1: Table S3). Finally, considering the positive association between low TyG index (≤ 8.80) and AF incidence after adjustments, we further performed sensitivity analyses between low TyG index and AF incidence stratified by different subgroups, including age, race, BMI, history of hypertension, non-diabetes and LDL-C level. After full adjustments, the positive association between low TyG index and AF incidence still existed (Additional file 1: Fig. S1).

## Discussion

### Major findings

Based on the longitudinal cohort of ARIC, we found a gradually rising risk of AF in patients without known CVDs with TyG levels after a median follow-up of 24.26 years. However, the full adjusted incidence of AF showed a U-shaped trend with a TyG level, with a nadir at a TyG level of approximately 8.80–9.20. Multivariable-adjusted Cox analysis and exposure-effect analysis further confirmed the U-shaped association. In a further sex-specific analysis, a U-shaped association between the TyG index and AF incidence still existed in females but not in males. Taken together, this study suggests a U-shaped association between the TyG index and AF incidence in people without known CVDs.

HOMA-IR is previously considered as the gold standard for IR. Recently, as an expected new estimating model for IR, the TyG index has been extensively investigated and approved with the strength of convenience compared to HOMA-IR^11^. Guerrero-Romero et al. found high sensitivity (96.5%) and specificity (85.0%) of the TyG index for recognizing IR [48]. The significant association between the TyG index and an increased risk of CVDs and mortality has been extensively explored [24, 30, 49–52], regardless of diabetes status. However, no studies have explored the association of the TyG index with AF in the general population.

Hence, it is not surprising that the higher TyG index is associated with an increased risk of AF in the present study. A positive association between the TyG index and AF risk has been observed in certain patients with established CVDs. In patients with hypertrophic obstructive cardiomyopathy undergoing septal myectomy and myocardial infarction receiving percutaneous coronary intervention, the TyG index showed moderate predictive ability for postoperative AF incidence [53, 54]. However, we did not assess the predictive ability of the addition of TyG in the existing risk scores for AF incidence, such as the Framingham Heart Study (FHS) score, the ARIC score, and the Cohorts for Heart and Aging Research in Genomic Epidemiology Atrial Fibrillation (CHARGE-AF) score, Thrombolysis in Myocardial Infarction 48 (TIMI-AF) and C2HEST [55–59], which may be further studied.

We showed that the crude rate of AF incidence increased with the TyG index. However, after full adjustments, we found a U-shaped association of the TyG index with AF incidence. Further sensitivity analyses basing individuals with low TyG index (≤ 8.80) among different subgroups including age, race, BMI, history of hypertension, nondiabetes, and LDL-C level showed the negative association between low TyG index and AF incidence still existed. Several potential mechanisms may be responsible. A strong association between a high TyG index and an increased risk of developing AF may be due to IR. IR in the liver is caused by impaired insulin metabolism influencing glucose metabolism and enhancing insulin-mediated lipogenesis to cause hyperglycemia [60]. Adipo-IR has been associated with β cell dysfunction, which begins in individuals with normal glucose tolerance [61]. Hyperglycemia and β cell impairment are both related to cardiomyocyte metabolism and cardiac function. All the above pathological changes may contribute to the incidence of atrial fibrillation. On the other hand, from the TyG index formula, we know low TyG index could cause by low triglyceride or low glucose levels. The positive association between a low TyG index and AF incidence may be explained by low fasting glucose. A recent systematic evaluation and meta-analysis showed that low fasting plasma glucose (< 4.0 mmol/L) was associated with an increased risk of future all-cause mortality, major cardiovascular events, and all-stroke and ischemic stroke in individuals without baseline cardiovascular disease or diabetes [62]. What’s more, a body of studies also found a low level of glucose showed a higher risk of diseases or events including AF, diabetes, stroke, major cardiovascular event, and all-cause death [63–65]. Low glucose may indicate abnormal nutritional and metabolic states which would increase the risk of cardiovascular events [66, 67]. As pancreatic cells are important coordinators in maintaining glucose, amino, and lipid homeostasis, we speculate the positive association between low TyG index and AF incidence may be related to pancreatic cellular dysfunction [68, 69]. Hence, hypoglycemia may be an indicator of cellular dysfunction. For further mechanism, the prolonged QTc interval enhanced adrenergic tone, and cardiac autonomic dysfunction caused by low plasma glucose could increase the risk of arrhythmia [70–72].

### Sex difference

Sex is a well-known modifier for the incidence and development of AF. Our previous meta-analysis showed that a higher TyG index was associated with coronary artery disease/CVD, even after removing sex-unadjusted studies (HR = 1.59, 95% CI 1.21–2.09) [31]. We showed a different shape of dose–response association trend between the TyG index and AF incidence in different sexes. Similar to our study, a sex difference (P = 0.045) in the relationship between a high TyG index and subclinical atherosclerosis in nondiabetic patients was found: a significantly higher prevalence of subclinical atherosclerosis in the high TyG index group than in the low TyG index group (odds ratio [OR] = 1.51; 95% CI 1.01–2.26) was observed in nondiabetic females but not in nondiabetic males (OR = 0.83; 95% CI 1.56–1.23) [73]. Obesity is a well-known risk factor for AF incidence. Several studies showed metabolically healthy obesity were at increased risk for all-cause mortality and cardiovascular events compared with metabolically healthy normal-weight individuals, although with controversial results [74–77]. Study by Fauchier et al. found an elevated risk of AF exists in metabolically healthy obese individuals, while female sex may be a modifier, which indicats the possible different role of obesity and metabolism between sex [78]. Despite recent studies attempt to standardize the concept of metabolically healthy obesity, there is no unified definition of metabolically healthy obesity [79, 80]. According to a meta-analysis, insulin sensitivity (eg, euglycemic-hyperinsulinemic clamps, HOMA-IR) was regarded as an important component to define the metabolically healthy obese phenotype [81]. Our analyses of increased AF incidence with high TyG index reinforced the above results. Differences in the location of fat storage in men and women may play a role in the development of IR and diabetes [82, 83]. Women tend to store fat subcutaneously rather than viscerally [84]. As visceral fat is closely linked to insulin resistance, whereas subcutaneous fat may be protective, women may need to gain more weight and experience a greater decline in associated metabolic risk factors to attain the same level of visceral fat storage [85, 86]. This implies that sex differences in metabolic risk factors result from elevated blood glucose levels and diabetes and assessing regional adiposity by imaging to further define both of obesity and metabolically healthy obesity is appropriate [87, 88].Additionally, in rodent models, endogenous estrogens may play a role in higher insulin sensitivity in women [89]. In a large clinical study, menopausal hormone therapy in postmenopausal women improved insulin sensitivity through estrogen receptors in the liver, muscle, and adipose tissue [90, 91]. The protective effect of estrogen is lost when women are exposed to risk factors such as hypertension and hyperlipidemia [92, 93], which may cause a sex difference.

Diabetes is another vital coufounding. Several previous studies have suggested that increased IR is associated with an increased risk of cardiovascular events in patients without diabetes [94, 95]. In our sensitivity analysis in nondiabetic individuals, the shape of the TyG index and AF did not significantly change. Due to the limited sample size of T2DM patients in the present cohort (8.9%), the relationship between the TyG index and AF is not assessed. Further studies are needed to clarify the role of the TyG index on AF in the diabetic population.

### Strengths and limitations

Our study has several strengths. To our knowledge, we are the first to investigate the association between the TyG index and the incidence of AF in individuals without known CVDs. Second, our data are from a well-designed longitudinal cohort that included a large sample size and diverse populations with adequate follow-up. Third, we further investigated the sex-specific association of the TyG index with AF incidence and found that female sex may be a modifier. However, our study also had several limitations. First, our analysis is based on an observational cohort study. Several confounding factors, such as nutritional status, may have influenced our results. In our analysis, the TyG index is calculated based on the blood sample at baseline. However, considering the median follow-up of 24.26 years, a fluctuation of TyG index values during follow-up may provide a better insight into the risk of AF incidence. Second, the population in our study is Americans, and the generality in other populations needs to be further verified.

## Conclusion

A U-shaped association between the TyG index and the AF incidence is observed in Americans without known CVDs. Female sex may be a modifier in the association between the TyG index and AF incidence.

## Supplementary Information

**Additional file 1: Table S1.** Basic characteristics of participants categorized according to gender. **Table S2.** Cox proportional hazards analysis evaluating prognostic implication of categorical TyG index for AF. **Table S3.** Cox proportional hazards analysis evaluating prognostic implication of categorical TyG index for AF among non-diabetic individuals. **Figure S1.** Multivariable-adjusted hazard ratios for AF based on restricted cubic spines inage≤54 years,age>54 years, African American,Caucasian,BMI<25.0 kg/m²,BMI 25.0-29.9 kg/m²,BMI≥30 kg/m²,nondiabetes,nonhypertension,hypertension,LDL-C<4.1 mmol/L,LDL-C≥4.1 mmol/L. Models were adjusted for age, race, body mass index, smoking, drinking, low density lipoprotein-cholesterol, high density lipoprotein-cholesterol, use of cholesterol lowering medication, history of diabetes, hypertension, peripheral artery disease. Red line represents references for HRs, and area between dotted line represent 95% CI. Blue area represents the fraction of the population with different baseline TyG index. HR: hazard ratio, CI: confidence interval, TyG triglyceride–glucose, AF: atrial fibrillation, BMI: body mass index, LDL-C: low density lipoprotein-cholesterol.

## Data Availability

The datasets used and analyzed during the current study are available from the corresponding author on reasonable request.

## Abbreviations

ABI: Ankle brachial index
AF: Atrial fibrillation
aHR: Adjusted hazard ratio
ARIC: Atherosclerosis Risk in Communities
BMI: Body mass index
CI: Confidence interval
CVD: Cardiovascular disease
ECG: Electrocardiogram
HDL-C: High-density lipoprotein-cholesterol
HOMA: Homeostatic model assessment
IQRs: Interquartile ranges
IR: Insulin resistance
LDL-C: Low-density lipoprotein-cholesterol
OR: Odds ratio
PAD: Peripheral artery disease
SDs: Standard deviations
TyG: Triglyceride–glucose
T2DM: Type 2 diabetes mellitus
