# Supplementary material for: U-shaped association between the triglyceride–glucose index and atrial fibrillation incidence in a general population without known cardiovascular disease
Source: Cardiovasc Diabetol. 2023 May 18;22:118. doi: 10.1186/s12933-023-01777-9 (PMC10197258; doi:10.1186/s12933-023-01777-9)
Supplement: Supplementary file 1 — Additional file 1: Table S1. Basic characteristics of participants categorized according to gender. Table S2. Cox proportional hazards analysis evaluating prognostic implication of categorical TyG index for AF. Table S3. Cox proportional hazards analysis evaluating prognostic implication of categorical TyG index for AF among non-diabetic individuals. Figure S1. Multivariable-adjusted hazard ratios for AF based on restricted cubic spines inage≤54 years,age>54 years, African American,Caucasian,BMI<25.0 kg/m²,BMI 25.0-29.9 kg/m²,BMI≥30 kg/m²,nondiabetes,nonhypertension,hypertension,LDL-C<4.1 mmol/L,LDL-C≥4.1 mmol/L. Models were adjusted for age, race, body mass index, smoking, drinking, low density lipoprotein-cholesterol, high density lipoprotein-cholesterol, use of cholesterol lowering medication, history of diabetes, hypertension, peripheral artery disease. Red line represents references for HRs, and area between dotted line represent 95% CI. Blue area represents the fraction of the population with different baseline TyG index. HR: hazard ratio, CI: confidence interval, TyG triglyceride–glucose, AF: atrial fibrillation, BMI: body mass index, LDL-C: low density lipoprotein-cholesterol. [file 12933_2023_1777_MOESM1_ESM.docx]

**Table S1. Basic characteristics of participants categorized according to gender.**

| **Variables** | **Male** | **Female** | **P-value** |
| --- | --- | --- | --- |
| Age(years) | 54.44 ±5.75 | 53.73±5.71 | <0.001 |
| Sex, n (%male) | 5265 | 6586 | <0.001 |
| BMI (kg/m²) | 27.29 ±4.04 | 27.44 ±5.90 | 0.113 |
| **Grace, n (%)** |  |  | <0.001 |
| Caucasian | 4162 (79.1) | 4916 (74.6) |  |
| African American | 1103 (20.9) | 1670 (25.4) |  |
| **Smoking status, n (%)** |  |  | <0.001 |
| Current smoker | 1403 (26.6) | 1589 (24.1) |  |
| Previous smoker | 2315 (44.0) | 1463 (22.2) |  |
| Never smoked | 1547 (29.4) | 3534 (53.7) |  |
| Baecke Sport Activity Score* | 2.59±0.82 | 2.35±0.76 | <0.001 |
| **drinking, n (%)** |  |  | <0.001 |
| Current | 3498 (66.4) | 3403 (51.7) |  |
| Previous | 1083 (20.6) | 988 (15.0) |  |
| Never | 684 (13.0) | 2195 (33.3) |  |
| history of clinical DM, n (%) | 477 (9.1) | 582 (8.8) | 0.696 |
| History of hypertension | 1619 (30.8) | 2014 (30.6) | 0.857 |
| History of PAD | 135 (2.6) | 307 (4.7) | <0.001 |
| Glucose (mg/dL) | 106.31 ±27.59 | 104.03 ±33.34 | <0.001 |
| Triglycerides (mg/dL) | 131.03 ±67.02 | 116.62 ±59.54 | <0.001 |
| HDL-C (mmol/L) | 1.16 ±0.36 | 1.51 ±0.44 | <0.001 |
| LDL-C (mmol/L) | 3.60 ±0.95 | 3.51 ±1.04 | <0.001 |
| TyG index | 8.71 ±0.56 | 8.57 ±0.56 | <0.001 |
| Cholesterol lowering medication, n (%) | 113 (2.1) | 173 (2.6) | 0.102 |
| Glucose lowering medication**#**, n (%) | 151 (2.9) | 229 (3.5) | 0.309 |

BMI: Body-mass index, DM: diabetes mellitus, PAD: peripheral artery disease, HDL-C: high-density lipoprotein cholesterol, LDL-C: low-density lipoprotein cholesterol, TyG index: triglyceride-glucose index.

* Values available in 11813 participants, **#** Values available in 11844 participants.

**Table S2: Cox proportional hazards analysis evaluating prognostic implication of categorical TyG index for AF.**

| **TyG**  **index** | **AF event/Total** | **Person-years** | **Incidence rate**  **(Per 100 person-years)** | **Unadjusted HR**  **(95%CI)** | **P-value** | **Model1** | **P-value** | **Model2** | **P-value** |
| --- | --- | --- | --- | --- | --- | --- | --- | --- | --- |
| <8.80  8.80-9.20  >9.20 | 1118/7605  427/2477  380/1769 | 161815.85  51327.01  33765.13 | 0.69  0.83  1.13 | 0.81 (0.72, 0.91)  1(ref.)  1.42 (1.23, 1.63) | <0.001  1  <0.001 | 1.15 (1.02, 1.29)  1(ref.)  1.19 (1.03, 1.37) | 0.027  1  0.021 | 1.15 (1.02, 1.29)  1(ref.)  1.18 (1.02, 1.36) | 0.026  1  0.028 |

In model1, HR adjusted for age, gender, race, body mass index, smoking, drinking, low density lipoprotein-cholesterol, high density lipoprotein-cholesterol, use of cholesterol lowering medication, history of diabetes, hypertension, peripheral artery disease, physical activity. In model2, HR adjusted for model1+ glucose lowering medication. AF: atrial fibrillation, TyG index: triglyceride-glucose index, HR: hazard ratios, CI: confidence intervals.

**Table S3: Cox proportional hazards analysis evaluating prognostic implication of categorical TyG index for AF among non-diabetic individuals.**

| **TyG**  **index** | **AF event/Total** | **Person-years** | **Incidence rate**  **(Per 100 person-years)** | **Unadjusted HR**  **(95%CI)** | **P-value** | **Adjusted HR**  **(95%CI)** | **P-value** |
| --- | --- | --- | --- | --- | --- | --- | --- |
| <8.80  8.80-9.20  >9.20 | 1077/7370  386/2270  233/1152 | 157383.10  47411.47  23253.64 | 0.68  0.81  1.00 | 0.82 (0.73, 0.92)  1(ref.)  1.25 (1.07, 1.48) | 0.001  1  0.006 | 1.14 (1.01,1.29)  1(ref.)  1.12 (0.95,1.32) | 0.038  1  0.170 |

HR adjusted for age, gender, race, body mass index, smoking, drinking, low density lipoprotein-cholesterol, high density lipoprotein-cholesterol, use of cholesterol lowering medication, history of hypertension and peripheral artery disease. AF: atrial fibrillation, TyG index: triglyceride-glucose index, HR: hazard ratios, CI: confidence intervals.


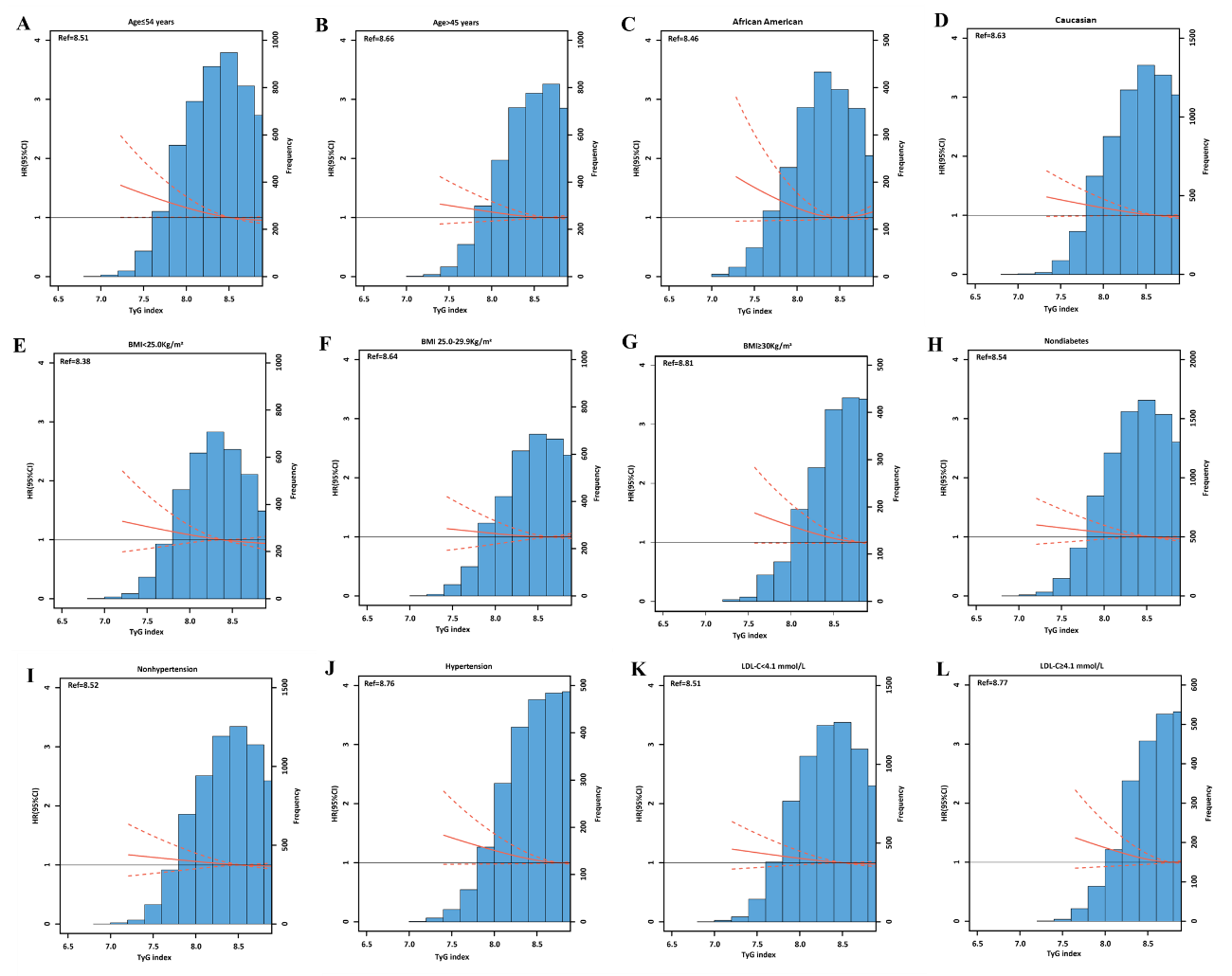


**FigureS1 Multivariable-adjusted hazard ratios for AF based on restricted cubic spines in (A) age≤54 years, (B) age>54 years, African American, (D) Caucasian, (E) BMI<25.0 kg/m², (F) BMI 25.0-29.9 kg/m², (G) BMI≥30 kg/m², (H) nondiabetes, (I) nonhypertension, (J) hypertension, (K) LDL-C<4.1 mmol/L, (L) LDL-C≥4.1 mmol/L.** Models were adjusted for age, race, body mass index, smoking, drinking, low density lipoprotein-cholesterol, high density lipoprotein-cholesterol, use of cholesterol lowering medication, history of diabetes, hypertension, peripheral artery disease. Red line represents references for HRs, and area between dotted line represent 95% CI. Blue area represents the fraction of the population with different baseline TyG index. HR: hazard ratio, CI: confidence interval, TyG triglyceride-glucose, AF: atrial fibrillation, BMI: body mass index, LDL-C: low density lipoprotein-cholesterol.
